# The Most-Cited Articles on Retinoblastoma: A Fifty-Year Perspective

**DOI:** 10.3390/vision7020033

**Published:** 2023-04-04

**Authors:** Rachel Shemesh, Hunter Sandler, Sarah Dichter, Ido Didi Fabian, Eedy Mezer, Tamara Wygnanski-Jaffe

**Affiliations:** 1 Sackler Faculty of Medicine, Tel-Aviv University, Tel-Aviv 6997801, Israel; 2 Sheba Medical Center, Goldschleger Eye Institute, Tel-Hashomer, Ramat Gan 52621, Israel; 3 Ruth and Bruce Rappaport Faculty of Medicine, Technion-Israel Institute of Technology, Haifa 3200003, Israel; 4 Department of Ophthalmology, Rambam Health Care Campus, Haifa 3109601, Israel

**Keywords:** retinoblastoma, bibliometrics, citations, treatment, chemotherapy

## Abstract

Purpose: To summarize the characteristics and trends of interest in retinoblastoma (Rb) in the last 50 years. Methods: The Web of Science Database was used to find all studies focused on Rb published from 1970 to 2018. The term “retinoblastoma” was used to search for the 100 most cited records. Results: The mean number of citations was 153.55 ± 88.9. The majority were from the United States (US) (*n* = 68). Drs. Shields authored 38% of the papers. The number of citations per year was positively correlated with the number of authors, r = 0.26 (*p* = 0.008). The number of patients was significantly associated with the number of citations per year (*p* = 0.012). Although papers on radiotherapy were the most common, publications about intra-arterial chemotherapy (IAC) were associated with 88.3% more citations per year (*p* = 0.031) and papers on intravenous chemotherapy (IVC) were associated with 40.3% more citations per year (*p*= 0.04). Review and meta-analysis studies had a higher median of citations (10.5) than interventional (6.4) or observational (5.2) studies. Conclusions: This study compiles a comprehensive analysis of the most-cited articles on Rb. Studies with a higher number of citations per year were associated with IAC, which emphasizes the significance of the advances in Rb treatments that allow for the saving of eyes and vision as well as lives. Review studies had more citations than observational or interventional studies. More citations were associated with a larger number of authors or more reported patients per paper. These findings highlight the importance of collaborations to achieve relevant, high-quality research of Rb.

## 1. Introduction

Retinoblastoma (Rb) is an aggressive eye cancer of the infant retina [1]. It is the most common intraocular malignancy of infancy and childhood, with an incidence of one case per 15,000–20,000 live births [2]. This pathology is caused by a mutation of the RB1 gene, and a person with an RB1 gene mutation has a greater than 90% chance of developing Rb and a higher risk of developing other types of cancer [3]. Diagnosis is made by fundoscopy and imaging, such as ultrasound and magnetic resonance imaging (MRI). The management of patients with Rb must consider the visual risk, the hereditary nature of the disease, and the life-threatening risk. Intra-arterial and intravitreal chemotherapy have appeared as promising methods to salvage eyes [4], yet enucleation is still often necessary [5]. In addition, adjuvant treatment may be recommended according to the histological risk factors [6]. Long-term follow-up and counseling regarding the risk of second primary tumors and transmission should be offered to these patients.

The purpose of this study was to identify the 100 most-cited publications on Rb throughout five decades and analyze the trends of interest in this field, providing a historical overview of the developments in the diagnosis, treatment, and follow-up of Rb patients.

## 2. Methods

The Web of Science Database was searched by an experienced medical librarian (SD) for the keyword “retinoblastoma” in the title, excluding the terms “protein” and “gene,” when searching for publications in peer-reviewed journals to create the list of the 100 most frequently cited clinical articles on Rb. The search included papers in all languages published between 1970 and 2018. This search was conducted in December 2021. We chose 1970 as the first year to provide a retrospective overview of the last 48 years, and 2018 was selected as the last year to allow enough time from when the article was published until it was cited. The papers were reviewed by 5 authors (RS, HS, EM, IDF and TWJ). Data parameters recorded from each paper are presented in Table 1.

### Statistical Analysis

Categorical variables were summarized as frequency and percentage. Continuous variables were summarized or reported as medians and interquartile ranges. The association between the number of citations per year and other continuous variables was evaluated using Spearman’s correlation coefficient. While the number of citations per year was compared between categories using the Kruskal–Wallis test or the Mann–Whitney test. The multivariable analysis included all variables that were significantly associated in the univariable analysis. The dependent variables and the number of participants were transformed using natural logs before applying linear regression. The linear regression was performed using the backward method. All statistical tests were two-sided, and *p* < 0.05 was considered statistically significant. The SPSS software was used (IBM SPSS Statistics for Windows, version 25, Armnok, NY, USA, 2017).

To account for the age of the article and number of citations, the number of citations of each article was divided by the number of years since publication to solve for “number of citations per year”.

## 3. Results

**Figure 1:**
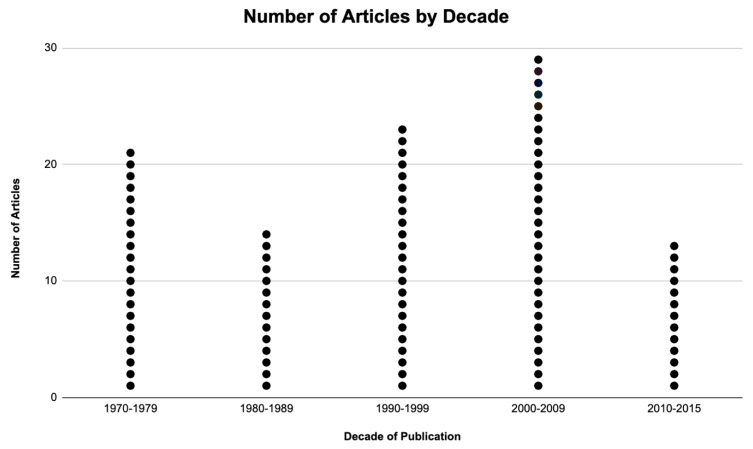
Time distribution of 100 top–cited articles on Retinoblastoma. Each publication is displayed as a dot, demonstrating the clustering of publications over time. Most articles were published in the years 2000–2009 (29%, *n*  =  29). The most recent year of publication of papers on the list was 2015.

The 100 most cited papers on Rb from the years 1970 to 2018 were compiled (Supplementary Table S1). The mean number of citations of a paper was 153.55 ± 88.9 (median 118, range 84–462). After a decline in the number of highly cited papers in the 1980s, the number of publications continued to rise over the next two decades, only to dip again in the 2010s (Figure 1). The decade with the most citations was the 2000s (29%, *n*  =  29). The papers were published in 38 different journals. Twenty-seven were American, seven British, four European, and two Asian journals. The leading journals were Ophthalmology (*n* = 14), the British Journal of Ophthalmology (*n* = 11), JAMA Ophthalmology (*n* = 10), the Journal of Clinical Oncology (*n* = 7), the American Journal of Ophthalmology (*n* = 5), Cancer (*n* = 4), the American Journal of Diseases in Children (*n* = 3), the Proceedings of the National Academy of Sciences (*n* = 3), and the Current Opinion in Ophthalmology (*n* = 3). Eleven other journals had two publications, and all other journals had one publication. All articles were published in a Q1 journal except for six and all were written in English. The papers’ mean impact factor was 10.95± 17.14 (median value 4.58, range 0.98–91.24).

### 3.1. Authorship

The first authors of the papers were affiliated with institutions in 13 different countries. The majority were from the United States (US) (*n* = 68), the United Kingdom (*n* = 7), and the Netherlands (*n* = 7). In all parameters, the last author affiliations were similar to the first author affiliations but varied with a total of 16 different country affiliations. Most of the last authors were from the United States (US) (*n* = 61), the Netherlands (*n* = 7), and the United Kingdom (*n* = 6). The Wills Eye Hospital and Thomas Jefferson University were the origins of the greatest number of articles on the list (*n* = 21), the New York Hospital-Cornell Medical Center, New York, NY, was second (*n* = 7), and four or fewer papers originated in the rest of the medical institutions (Table 2). Drs. Carol L. and Jerry A. Shields from the Wills Eye Hospital, USA, published the greatest number of articles in the list (*n* = 19 each), followed by Dr. D.H. Abramson from the Memorial Sloan Kettering Cancer Center, USA (*n* = 14), while all others authored eight or fewer papers (Table 3). All the papers authored by the Shields were published between the 1980s and 2010s and all discussed treatment intervention options, with 17 papers discussing radiation therapy, 16 papers discussing intravenous chemotherapy and/or enucleation, 10 papers discussing chemo reduction with focal consolidation, and 6 papers discussing vitreous body injections and/or intra-arterial chemotherapy. Additionally, the topic of metastasis was discussed in 11 of these publications. The type of study varied between observational (*n* = 6), interventional (*n* = 7), and review/meta-analysis (*n* = 7).

When the affiliations of the first and last authors of the 100 most cited papers were compared by continent, most authors were affiliated with a North American institute (*n* = 70, 63). The mean number of citations per year was greatest when the first author was from North America (7.6 ± 5.5, median 6.2, range 1.9–24.3) or Europe (8.7 ± 7.1, median 4.4, range 1.8–33.4). The papers from Asia, Africa, and South America had the least citations and were grouped together for statistical analysis (6.9 ±7.0, median 7.0, range 2.1–10.9). 

The mean number of authors for each paper was 5.7 ± 5.3 (median 4, range 1–46). Using univariable analysis, there was a positive correlation between the number of citations per year and the number of authors, r = 0.26 (*p* = 0.008). Using the multivariate with backwards method, it was discovered that adding one author to each study increased the number of citations per year by 4.6% (*p* = 0.067).

### 3.2. Number of Patients

In 75 clinical papers, the number of patients analyzed varied, with a mean of 462.2 ± 732.4 patients (median 130, range 1–3544). When univariable analysis was performed, there was a positive correlation between the number of patients in the study and the number of citations per year, r = 0.23, (*p* = 0.048). When the multivariate with backwards method was performed, the number of patients was significantly associated with the number of citations per year (*p* = 0.012), and an increase of 1% in the number of patients in each study was found to increase the number of citations per year by 0.1% (*p* = 0.012).

### 3.3. Category

The articles were divided into three categories: observational, interventional, and review/meta-analysis. In total, 71% of the published papers were observational studies, 15% were interventional, and 14% were reviews. When comparing the article types, the review and meta-analysis studies had significantly higher citations per year (median 10.5, range 8.5–18.9) in comparison to the observational studies (median 5.2, range 2.8–9.0), (*p* = 0.001) and interventional studies (median 6.4, range 4.3–9.1), (*p* = 0.016). Fifty-eight papers were funded, and eight of those were multicenter studies.

### 3.4. Topics

The topics assessed in the papers were on the following subjects: treatment (*n* = 67), incidence (*n* = 40), genetics (*n* = 35), pathology (*n* = 33), metastasis (*n* = 33), secondary tumors (*n* = 31), classification system (*n* = 19), diagnosis/screening (*n* = 13), and trilateral Rb (*n* = 7). The topic of treatment was further divided into methods of treatment, including radiation (*n* = 59), surgery (*n* = 50), intravenous chemotherapy (*n*= 48), chemoreduction with focal consolidation (*n* = 16), intra-arterial chemotherapy (*n* = 9), and vitreous body injections (*n* = 7). When using the multivariate with backwards method, the papers about intravenous chemotherapy were associated with 40.3% more citations per year in comparison to the papers that did not include this topic (*p* = 0.04). In addition, the papers on the topic of intra-arterial chemotherapy were associated with 88.3% more citations per year in comparison to the papers that did not include this topic (*p* = 0.031). Yet, the papers on the topic of surgical treatment were associated with 34.5% fewer citations per year in comparison to the papers that did not include this topic (*p* = 0.009).

### 3.5. The Ten Most Cited Papers

In most parameters, the characteristics of the top ten most cited papers were consistent with the rest of the list. The countries of affiliation were six from the US, two from the United Kingdom, one from the Netherlands, and one from Finland. Drs. Shields authored one of the top ten most cited papers (7th place). Eight of the top ten papers were affiliated with universities, nine were affiliated with hospitals, and four were funded. The type of study varied, with eight observational studies, one interventional study, and one review and data analysis. The number of authors varied from one to nine on these papers. Six of the papers discussed incidence; five papers discussed genetics; two papers discussed the classification system for Rb; seven papers discussed treatment options; and four papers discussed secondary tumors. Of the papers that discussed treatment options, six included radiation, five included intravenous chemotherapy, one discussed chemoreduction with focal consolidation, and two discussed surgery treatment. The two most cited papers on the list were on the prevention of secondary tumors.

## 4. Discussion

In recent years, new therapies have emerged as promising methods to salvage lives, eyes, and vision. With the constant development of imaging and surgical techniques, an abundant literature has emerged, helping to improve the diagnosis, follow-up, and treatment of Rb patients. However, the volume of literature makes it difficult to identify the papers with significant contributions. The purpose of this study was to conduct the first bibliographic study that covered the top 100 cited articles on Rb and to gain insight into the trends, novelty, and quality of the published work in the last 50 years.

### 4.1. Authorship and Number of Patients

Two hundred and fifty to three hundred and fifty Rb cases are diagnosed each year in the USA [7] and the improvement in the survival of Rb patients has been faster than that of any other cancer in children or adults in the USA [8]. Moreover, in the Western world, 99% of children will survive Rb and over 90% will retain normal vision in at least one eye [9]. This is reflected in our analysis as most of the pioneering research work on the diagnosis, treatment, and follow-up of Rb patients was conducted in North America. Additionally, when the first author was North American, the mean number of citations per year was the greatest.

The study of Girdler et al. has identified many global collaborations involving patient referrals, consultations, and twinning/capacity building, demonstrating an extensive effort to reduce retinoblastoma mortality [10]. Such initiatives explain the significant positive correlation between the number of citations per year and the number of authors found in our study. The number of patients was also significantly associated with the number of citations per year, as an increase of 1% in the number of patients in each study was found to increase the number of citations per year by 0.1%. This emphasizes the importance of a large cohort of patients, which can be more easily achieved by collaborations between countries [11]. 

All the papers on our list authored by the Shields were published between the years of 1981 and 2015. A nearly equal distribution was found between the number of papers published in the 1990s (*n* = 6), 2000s (*n* = 7), and 2010s (*n* = 6). This indicates the constant contribution and relevance the Shields have had in the field of Rb research. The type of study varied with six observational, seven interventional, and seven review articles, showing the array of study methods that the Shields implement. All 19 of the respected publications by Dr. Carol and Dr. Jerry Shields included the topic of treatment interventions and covered every treatment option. This aligns with the general trend in our list of treatment intervention being the most recurring topic (*n* = 67), possibly due to the recent advances made in Rb treatment. The paper authored by the Shields in the top ten publications discussed Rb classification and intravenous chemotherapy, following the trend in our list: the papers that discuss intravenous chemotherapy were associated with 40.3% more citations a year. Lastly, another main topic discussed in many of the Shields’ publications was metastases (*n* = 11). Like the increase in publications on secondary tumors from radiotherapy, as survival rates for Rb patients increase, more metastases are being observed.

### 4.2. Category

The review and meta-analysis papers had significantly more citations per year in comparison to the observational studies and interventional studies. Meta-analysis includes the process of extracting quantitative data from many papers, thus enabling the analysis of a large amount of data from many studies conducted in many centers, which summarizes important results on this rare disease. Such studies allow for a new understanding of the evolution of Rb treatment and follow-up, which have undergone considerable change during the last few years [12,13,14]. One example of this kind of evolution is described in a well cited review by the International Retinoblastoma Staging Working Group, which is composed of participants from 24 countries on four continents. This work discussed: the staging and tissue handling guidelines to reach a consensus on adequate processing, establishing definitions of histopathologic risk factors, and the reporting of enucleated eyes with Rb [15]. 

### 4.3. Topics

In our study, we found that the articles on the top of the list were published on a variety of topics, consisting of treatment (*n* = 67), incidence (*n* = 40), genetics (*n* = 35), pathology (*n* = 33), metastasis (*n* = 33), secondary tumors (*n* = 31), classification system (*n* = 19), diagnosis/screening (*n* = 13), and trilateral Rb (*n* = 7). Majority of the papers were on the topic of treatment. This could be explained by the advances made in Rb treatments in recent years. Intra-arterial chemotherapy is one of these advances. This method involves a single-agent injection into the ophthalmic artery under careful neuro-interventional guidance [16]. This pioneering therapy can be useful for eyes that fail standard treatments or as a primary treatment [7]. The systematic review of Yousef et al. [17] showed that across all published literature for intra-arterial chemotherapy, globe salvage was achieved for 502 (66%) of all eyes and 57% of eyes with advanced disease. In our study, the papers on the topic of intra-arterial chemotherapy were associated with 88.3% more citations per year in comparison to the papers that did not include this topic (*p* = 0.031). This finding emphasizes the great impact of this treatment on the Rb medical field, offering a promising new treatment associated with high rates of globe salvage. The primary goals of Rb treatment are patient survival, eye protection, and, lastly, visual function. As a result, primary enucleation is still used in some cases. In our study, the papers on the topic of surgical treatment were associated with 34.5% fewer citations per year, demonstrating this trend. Nevertheless, the papers about intravenous chemotherapy were associated with 40.3% more citations per year. This may be because many times a combination of treatments is used, including intravenous chemotherapy as induction or adjuvant therapy, explaining the abundance of citations on the subject [18,19].

### 4.4. The Ten Most Cited Papers

Like the discussion mentioned above, the 10 most cited papers on Rb were mostly affiliated with the US (60%). The majority were observational studies (80%) and the number of authors varied from one to nine on these papers. Treatment options were discussed in 70% of these papers, including radiation (60%) and intravenous chemotherapy (50%). This could be explained by the high concern of secondary tumors post radiation treatment in Rb patients [20,21]. The two most cited papers on the list were on secondary tumors, highlighting the long-term ramifications of using radiation as a treatment for Rb patients and the need for follow-up into later life [20,21]. These studies are becoming more relevant as Rb patient survival increases and secondary tumors develop [22]. Furthermore, advances in radiology are being made to prevent high doses of radiation in these patients. Monroe et al. studied the imaging techniques and radiation exposure of Rb patients treated with intra-arterial chemotherapy and showed radiation exposure during treatment can be dramatically reduced without affecting technical success or safety [23].

### 4.5. Publication Time

It may be assumed that the true impact of a study cannot be evaluated for at least two decades from its publication [24]. In our study, a continued increase in the number of published papers was observed from the 1980s until the 2010s, when it began to decline. This can possibly be attributed to the advent of the internet, which has allowed for global communication and collaboration. This has been a key factor in determining new classification systems [25,26], new treatment techniques [27,28,29], and evaluating the outcomes of these patients all over the globe [30,31]. In the present list, only 13 articles were published after the year 2010. This could be due to insufficient time passing since their publication for citations to accumulate. Some relevant articles published prior to the decade of the 1970s could have been omitted as there are no electronic versions of those papers.

### 4.6. Study Limitations

The present study has several limitations. First, the number of citations provided by the Web of Science might not be inclusive of published publications. Other search means may have produced a different list of the most cited articles on Rb. Although the Web of Science is known to be a trusted publisher-independent global citation database, using a few search engines could enable a better validation of the 100 most-cited papers list. Second, examining the number of citations of an article is a limited and objective measure of the relevance of a study. Third, recently published studies were not included in our analysis. These studies may have greatly influenced the field of Rb but have not yet generated many citations as they have only recently been published. In this study, 2018 was determined to be the last year of publications to allow enough time from when the article was published until it was cited.

In conclusion, this study demonstrated that most papers published in the US were by authors who were affiliated with US hospitals. The importance of conducting novel and significant research in the field of Rb can be seen as the improvement in the survival of Rb patients in the US has been faster than any other cancer in children or adults in the USA and can be at least partially attributed to the pioneering research work conducted in North America. The Shields, who are experts in the field of Rb, authored a remarkable 19% of the papers. Although most citations were observational, review and meta-analysis papers had more citations on average. This is likely because these study methods involved distributing large quantities of data from many papers to allow for a new understanding of the evolution of Rb treatment and follow-up, which have undergone considerable changes during the last few years. The treatment of Rb was the focus of the most cited papers. The treatment of Rb was the focus of the most cited papers on Rb. While most papers on the list dealt with radiotherapy, the papers on intravenous and intra-arterial chemotherapy were associated with more citations per year, had the greatest impact on citation rank, and were associated with the most citations per year. This emphasizes the significance of recent advances in RB treatments, which allow for the saving of eyes and vision as well as lives. Additionally, the papers with more authors or a larger number of subjects were also associated with more citations, as these papers were collaborations among many centers and even many countries, with higher external and internal validity studies. The two most cited articles on the list, with more than 400 citations each, were on secondary tumors in Rb patients, emphasizing the importance of reducing radiation exposure in these patients and a lifelong follow-up. In conclusion, the current study analyzed the 100 most cited articles on Rb in the last 50 years according to their authors, institutions, countries, and journals, to outline the cause of their important contributions to this field.

## Figures and Tables

**Table 1:** Data that was recorded from each paper.

Data	Details
Number of citations	Includes the mean number of citations per year since publication (The Web of Science database was searched between the years 1970 and 2018).
Journal details	The journal name, impact factor, quartile, the year of publication, and the publication language
Authors details	The names and number of authors, the country and continent of origin of the first and last authors, and the affiliations of the first and last authors
Type and methodology of article	The type of article based on the different types of articles listed in JAMA Ophthalmology Journal*, the type of research methodology, and the type of study (single or multicenter study)
Number of participants	The number of patients included (when applicable)
Topics	Incidence, genetics, classification system, pathology, diagnosis and screening, treatment, trilateral retinoblastoma, secondary tumors, and metastasis
Funding of study	External funding for the research

**Table 2:** Number of articles from an institution affiliated with an author with 2 or more publications.

Name of Institute	Number of Articles
Wills Eye Institute and Thomas Jefferson University, Philadelphia, PA, USA	21
New York Hospital-Cornell Medical Center, New York, NY, USA	7
Armed Forces Institute of Pathology, Washington, DC, USA	4
Memorial Sloan Kettering, New York, NY, USA	4
Hospital JP Garrahan, Buenos Aires, Argentina	4
University of Oxford, Oxford, England	4
Helsinki University Central Hospital, Helsinki, Finland	3
Children’s Hospital, Los Angeles, CA, USA	3
University of Texas, Houston, TX, USA	3
University of Southern California, Los Angeles, CA, USA	3
National Cancer Institute, Bethesda, MD, USA	3
Institut Curie, Paris, France	3
Harvard University, Cambridge, MA, USA	3
VU University Medical Center, Amsterdam, The Netherlands	2
Cleveland Clinic, Cleveland, OH, USA	2
The George Washington University Medical Center, Washington, DC, USA	2
Vrije Universiteit, Amsterdam, The Netherlands	2
University of Toronto, Toronto, ON, Canada	2
Free University, Amsterdam, The Netherlands	2

**Table 3:** Contributing authors with 3 or more publications: initials, last name, and credentials.

Author Name	Number of Publications	Author Name	Number of Publications
J.A., Shields MD	19	C.M., Frank MD	3
C.L., Shields MD	19	A.G. Knudson MD, PhD	3
D.H., Abramson MD	14	L., Desjardins MD	3
A., Meadows MD	8	S.G., Honavar MD	3
C., Rodriguez-Galindo MD	7	H., Demirci MD	3
G., Chantada MD	7	T., Kivelä MD	3
I., Dunkel, MD	6	E., Schvartzman MD, PhD	3
S.M., Imhof MD, PhD	5	M.G., Wilson MD	3
J., Cater PhD	5	C.B.G., Antoneli MD	3
A.D., Singh MD	5	M.O., Ts’O MD	3
A.C., Moll MD, PhD	5	L.E., Zimmerman MD	3
G.J., Draper MD	4	D.J., Kuik M.Sc	3
P., De Potter MD	4	E. Quintana DO	3
B., Haik MD	4	B., Gallie MD	3
B.M., Sanders MD	4	J., Michon MD	3
F., Doz MD	4	M.T., De Davila MD	3
K. E.W.P, Tan MD	4	J.W., Koten MD, PhD	3
R.M., Ellsworth MD	4	W., Den-Otter MD	3
A.L., Murphree MD	3		

## Data Availability

Data is contained within the article.

## Supplementary Materials

The following supporting information can be downloaded at: https://www.mdpi.com/article/10.3390/vision7020033/s1, Table S1: 100 most cited Rb papers published from 1970–2018.
