# Supplementary material for: The Most-Cited Articles on Retinoblastoma: A Fifty-Year Perspective
Source: Vision (Basel). 2023 Apr 4;7(2):33. doi: 10.3390/vision7020033 (PMC10123740; doi:10.3390/vision7020033)
Supplement: Supplementary file 1 [file vision-07-00033-s001.zip › vision-2188339-supplementary.pdf]

Table S1. The 100 most frequently cited papers on Retinoblastoma in descending order.

Article Position	No. of Citations	Author's First Name	Author's Last Name	Journal Name	Publication Year	Original Title
1	462	Draper	Kingston	British journal of cancer	1986	Second Primary Neoplasms in Patients with Retinoblastoma
2	457	Wong	Frederick	JAMA	1997	Cancer incidence after retinoblastoma - Radiation dose and sarcoma risk
3	455	Yunis	Ramsay	American journal of diseases of children	1978	Retinoblastoma and Subband Deletion of Chromosome 13
4	407	Kivela		British Journal of Ophthalmology	2009	The Epidemiological Challenge of the Most Frequent Eye Cancer: Retinoblastoma, an Issue of Birth and Death
5	380	Abramson	Tung	Ophthalmology	1984	Second Nonocular Tumors in Retinoblastoma Survivors: Are They Radiation-induced?
6	360	Murphree		Ophthalmology clinics of North America	2005	Intraocular Retinoblastoma: The Case for a New Group Classification
7	358	Shields	Shields	Ophthalmology	2006	The International Classification of Retinoblastoma Predicts Chemoreduction Success
8	333	Murphree	Gomer	JAMA ophthalmology	1996	Chemotherapy plus local treatment in the management of intraocular retinoblastoma
9	309	Marees	Moll	European Journal of Cancer	2009	Cancer Mortality in Long-term Survivors of Retinoblastoma
10	302	Knudson Jr	Brown	Proceedings of the National Academy of Sciences	1975	Mutation and Childhood Cancer - Probabilistic Model for Incidence of Retinoblastoma
11	276	Knudson Jr	Hill	New England Journal of Medicine	1976	Chromosomal Deletion and Retinoblastoma
12	266	Shields	Maris	JAMA ophthalmology	1996	Chemoreduction in the Initial Management of Intraocular Retinoblastoma
13	250	Tombran-Tink	Johnson		1989	Neuronal Differentiation of Retinoblastoma Cells Induced by Medium Conditioned by Human Rope Cells

				Investigative ophthalmology & visual science		
14	213	Kingston	Plowman	JAMA ophthalmology	1996	Results of Combined Chemotherapy and Radiotherapy for Advanced Intraocular Retinoblastoma
15	208	Broaddus	Singh	British Journal of Ophthalmology	2009	Incidence of Retinoblastoma in the USA: 1975-2004
16	208	Abramson	Frank	Ophthalmology	1998	Second Nonocular Tumors in Survivors of Bilateral Retinoblastoma - a Possible Age Effect on Radiation-related Risk
17	208	Zampetti-Bosseler	Scott	International Journal of Radiation Biology and Related Studies in Physics, Chemistry and Medicine	1981	Cell-death, Chromosome-damage and Mitotic Delay in Normal Human, Ataxia Telangiectasia and Retinoblastoma Fibroblasts After X-irradiation
18	197	Jensen	Miller	New England Journal of Medicine	1971	Retinoblastoma - Epidemiologic Characteristics
19	194	Shields	Shields	Current opinion in ophthalmology	2010	Retinoblastoma Management: Advances in Enucleation, Intravenous Chemoreduction, and Intra-arterial Chemotherapy
20	192	Knudson Jr		Seminars in oncology	1978	Retinoblastoma- prototypic hereditary neoplasm
21	191	Friedman	Meadows	Journal of Clinical Oncology	2000	Chemoreduction and Local Ophthalmic Therapy for Intraocular Retinoblastoma
22	187	McFall	Makadon	Cancer research	1977	Characterization of a New Continuous Cell Line Derived from a Human Retinoblastoma
23	185	Yamane	Mohri	International journal of clinical oncology	2004	The Technique of Ophthalmic Arterial Infusion Therapy for Patients with Intraocular Retinoblastoma
24	179	Khelfaoui	Vielh	Cancer	1996	Histopathologic Risk Factors in Retinoblastoma - a Retrospective Study of 172 Patients Treated in a Single Institute
25	173	Lee	Smith	International Journal of Radiation Oncology Biology Physics	2005	Treatment Planning with Protons for Pediatric Retinoblastoma, Medulloblastoma, and Pelvic Sarcoma
26	170	Simpson	Dendale	Brachytherapy	2014	The American Brachytherapy Society Consensus Guidelines for Plaque Brachytherapy of Uveal Melanoma and Retinoblastoma

27	169	Shields	De-Potter	Cancer	1994	Optic-nerve Invasion of Retinoblastoma - Metastatic Potential and Clinical Risk Factors
28	164	Honavar	Shields	JAMA ophthalmology	2002	Postenucleation Adjuvant Therapy in High-risk Retinoblastoma
29	160	Shields	De-Potter	British journal of ophthalmology	1993	Choroidal Invasion of Retinoblastoma - Metastatic Potential and Clinical Risk Factors
30	158	Shields	Naduvilat h	American journal of ophthalmology	2002	Chemoreduction Plus Focal Therapy for Retinoblastoma: Factors Predictive of Need for Treatment with External Beam Radiotherapy or Enucleation
31	150	Kivelä	Kivelä, Tero	Journal of Clinical Oncology	1999	Trilateral Retinoblastoma: A Meta-analysis of Hereditary Retinoblastoma Associated with Primary Ectopic Intracranial Retinoblastoma
32	146	Abramson	Dunkel	Ophthalmology	1999	A Phase I/II Study of Subconjunctival Carboplatin for Intraocular Retinoblastoma
33	146	Kopelman	Rosenberg	Ophthalmology	1987	Multivariate-analysis of Risk-factors for Metastasis in Retinoblastoma Treated by Enucleation
34	142	Chintagump ala	Hurwitz	The Oncologist	2007	Retinoblastoma: Review of Current Management
35	141	Gombos	Leal	Ophthalmology	2007	Secondary Acute Myelogenous Leukemia in Patients with Retinoblastoma - Is Chemotherapy a Factor
36	141	Abramson	Boyd III	The Journal of pediatrics	1998	Presenting Signs of Retinoblastoma
37	140	Shields	Shields	Current opinion in ophthalmology	2006	Basic Understanding of Current Classification and Management of
38	136	Abramson	Chantada	JAMA ophthalmology	2015	Treatment of Retino Blastionma in 2015 Agreement and Disagreement
39	135	Chantada	Schvartzm an	Pediatric blood & cancer	2006	A Proposal for an International Retinoblastoma Staging System
40	133	Shields	Meadows	Ophthalmology	1997	Combined Chemoreduction and Adjuvant Treatment for Intraocular Retinoblastoma
41	131	Sastre	Chevez-Barrios	Archives of pathology & laboratory medicine	2009	Proceedings of the Consensus Meetings from the International Retinoblastoma Staging Working

						Group on the Pathology Guidelines for the Examination of Enucleated Eyes and Evaluation of Prognostic Risk Factors in Retinoblastoma
42	129	Sanders	Kingston	British Journal of Ophthalmology	1988	Retinoblastoma in Great-Britain 1969-80 - Incidence, Treatment, and Survival
43	126	Lalande	Latt	Cancer	1984	Isolation of Human-chromosome 13-specific DNA-Sequences Cloned From Flow Sorted Chromosomes and Potentially Linked to the Retinoblastoma Locus
44	124	Tamboli	Hrom	JAMA ophthalmology	1990	The Incidence of Retinoblastoma in the United States - 1974 Through 1985
45	122	Hethcote	Knudson Jr	Proceedings of the National Academy of Sciences	1978	Model for Incidence of Embryonal Cancers - Application to Retinoblastoma
46	121	Broaddus	Singh	British Journal of Ophthalmology	2009	Survival with Retinoblastoma in the USA: 1975-2004
47	121	Draper	Hawkins	British journal of cancer	1992	Patterns of Risk of Hereditary Retinoblastoma and Applications to Genetic- Counseling
48	119	Canturk	Rodriguez -Galindo	British Journal of Ophthalmology	2010	Survival of Retinoblastoma in Less-developed Countries Impact of Socioeconomic and Health- Related Indicators
49	118	Ts'O	Zimmerm an	American journal of ophthalmology	1970	The Nature of Retinoblastoma. II. Photoreceptor Differentiation: an Electron Microscopic Study.
50	118	Abramson	Zimmerm an	Ophthalmology	1976	Non-ocular Cancer in Retinoblastoma Survivors
51	116	Rodriguez-Galindo	Kun	Journal of Clinical Oncology	2003	Treatment of Intraocular Retinoblastoma with Vincristine and Carboplatin
52	115	Moll	Boers	Ophthalmology	2001	Second Primary Tumors in Hereditary Retinoblastoma: a Register-based Study, 1945-1997 - Is There an Age Effect on Radiation-related Risk?
53	114	Shields	Shields	JAMA ophthalmology	2011	Intra-arterial Chemotherapy for Retinoblastoma Report No. 2, Treatment Complications
54	114	Pendergrass	Davis	JAMA ophthalmology	1980	Incidence of Retinoblastoma in the United-States

55	114	Ts' O	Fine	American journal of ophthalmology	1970	The Nature of Retinoblastoma. I. Photoreceptor Differentiation: a Clinical and Histopathologic Study.
56	113	Chantada	Schvartzman	Archives of disease in childhood	1999	Late Diagnosis of Retinoblastoma in a Developing Country
57	112	Shields	Micaily	Ophthalmology	2001	Plaque Radiotherapy for Retinoblastoma - Long-term Tumor Control and Treatment Complications in 208 Tumors
58	111	Kaliki	Eagle Jr.	Ophthalmology	2013	High-Risk Retinoblastoma Based on International Classification of Retinoblastoma: Analysis of 519 Enucleated Eyes
59	111	Abramson	Ranjithan	Pediatrics	2003	Screening for Retinoblastoma: Presenting Signs as Prognosticators of Patient and Ocular Survival
60	110	Abramson	Ellsworth	American Journal of Ophthalmology	1979	Second Tumors in Non-irradiated Bilateral Retinoblastoma
61	109	Shields	Shields	Eye	2013	Retinoblastoma Frontiers with Intravenous, Intra-arterial, Periocular, and Intravitreal chemotherapy
62	109	Shields	Naduvilath	JAMA ophthalmology	2002	Factors Predictive of Recurrence of Retinal Tumors, Vitreous Seeds, and Subretinal Seeds Following Chemoreduction for Retinoblastoma
63	109	Messmer	Sauerwein	Ophthalmology	1991	Risk-Factors for Metastases in Patients with Retinoblastoma
64	108	Moll	Tan	British Journal of Ophthalmology	1997	Incidence and Survival of Retinoblastoma in the Netherlands: a Register Based Study 1862-1995
65	108	Nagasawa	Little	Mutation Research/Fundamental and Molecular Mechanisms of Mutagenesis	1983	Comparison of Kinetics of X-ray-induced Cell Killing in Normal, Ataxia Telangiectasia and Hereditary Retinoblastoma Fibroblasts
66	107	Devesa		American journal of ophthalmology	1975	Incidence of Retinoblastoma
67	107	Jakobiec	Danis	Cancer	1977	Retinoblastoma and Intracranial Malignancy
68	106	Shields	Shields	Journal of Pediatric Ophthalmology & Strabismus	1999	Recent Developments in the Management of Retinoblastoma

69	105	Leander	Haik	Pediatric blood & cancer	2007	Impact of an Education Program on Late Diagnosis of Retinoblastoma in Honduras
70	103	Shields	Shields	JAMA ophthalmology	1999	Thermotherapy for Retinoblastoma
71	102	Squire	Phillips	Human genetics	1985	A Detailed Analysis of Chromosomal Changes in Heritable and Non-heritable Retinoblastoma
72	102	Benedict	Murphree	Cancer genetics	1983	Nonrandom Chromosomal Changes in Untreated Retinoblastomas
73	100	de Graaf	Brisse	Pediatric radiology	2012	Guidelines for Imaging Retinoblastoma: Imaging Principles and MRI Standardization
74	100	Eagle Jr		Archives of pathology & laboratory medicine	2009	High-risk Features and Tumor Differentiation in Retinoblastoma a Retrospective Histopathologic Study
75	99	Magramm	Ellsworth	Ophthalmology	1989	Optic-nerve Involvement in Retinoblastoma
76	96	Shields	Shields	Current Opinion in Ophthalmology	2014	Targeted Retinoblastoma Management: When to Use Intravenous, Intra-arterial, Periocular, and Intravitreal Chemotherapy
77	96	Wilson	Fujimoto	American journal of human genetics	1973	Retinoblastoma and D-chromosome Deletions
78	95	Lennox	Sanders	British Journal of Medicine	1975	Retinoblastoma - Study of Natural-history and Prognosis of 268 Cases
79	94	Abramson		Investigative ophthalmology & visual science	2005	Retinoblastoma in the 20th Century: Past Success and Future Challenges - The Weisenfeld Lecture
80	94	Chantada	Abramson	British journal of ophthalmology	2004	Retinoblastoma Patients with High Risk Ocular Pathological Features: Who Needs Adjuvant Therapy
81	93	Abramson	Gobin	British journal of ophthalmology	2012	Intra-arterial Chemotherapy for Retinoblastoma in Eyes with Vitreous and/or Subretinal Seeding: 2-year Results
82	93	Weichselbaum	Little	Proceedings of the National Academy of Sciences	1978	X-ray Sensitivity of Diploid Fibroblasts from Patients with Hereditary or Sporadic Retinoblastoma
83	92	Shields	Augsburger	Survey of ophthalmology	1981	Current Approaches to the Diagnosis and Management of Retinoblastoma
84	91	Beck	Munier	Journal of Clinical Oncology	2000	First-line Chemotherapy with Local Treatment Can Prevent External-beam Irradiation and Enucleation in Low-stage Intraocular Retinoblastoma

85	91	Bedford	Macfaul	The British journal of ophthalmology	1971	Retinoblastoma - Study of 139 Cases
86	89	Sethi	MacDonald	Cancer	2014	Second Nonocular Tumors Among Survivors of Retinoblastoma Treated with Contemporary Photon and Proton Radiotherapy
87	89	Qaddoumi	Rodriguez-Galindo	Journal of clinical oncology	2012	Carboplatin-Associated Ototoxicity in Children with Retinoblastoma
88	89	Namouni	Lutz	European journal of cancer	1997	High-dose Chemotherapy with Carboplatin, Etoposide Andcyclophosphamide Followed by a Haematopoietic Stem Cell Rescue in Patients with High-risk Retinoblastoma: a Sfop and Sfgm Study
89	89	Stannard	Sevel	British Journal of Ophthalmology	1979	Retinoblastoma - Correlation of Invasion of the Optic-nerve and Choroid with Prognosis and Metastases
90	89	Sparkes	Klisak	Science	1979	Retinoblastoma With 13q-chromosomal Deletion Associated with Maternal Paracentric Inversion of 13q
91	88	Uusitalo	O'Brien	JAMA ophthalmology	2001	Evaluation of Chemoprophylaxis in Patients with Unilateral Retinoblastoma with High-risk Features on Histopathologic Examination
92	88	Schvartzman	Manzitti	Journal of clinical oncology	1996	Results of a Stage-based Protocol for the Treatment of Retinoblastoma
93	87	Moll	Tan	International journal of cancer	1996	Second Primary Tumors in Patients with Hereditary Retinoblastoma: A Register-based Follow-up Study, 1945-1994
94	87	Doz	Vielh	Cancer	1994	The Role of Chemotherapy in Orbital Involvement of Retinoblastoma - the Experience of a Single Institution With 33 Patients
95	86	Yu	Kleinerman	Journal of the National Cancer Institute	2009	Cause-specific Mortality in Long-term Survivors of Retinoblastoma
96	86	DerKinderen	Otter	International journal of cancer	1988	Non-ocular Cancer in Patients with Hereditary Retinoblastoma and Their Relatives
97	86	Egbert	Rosenthal	JAMA ophthalmology	1978	Visual Results and Ocular Complications Following Radiotherapy for Retinoblastoma
98	85	Zhao	Gallie		2011	Pre-enucleation Chemotherapy for Eyes Severely Affected by Retinoblastoma Masks Risk of

				Journal of Clinical Oncology		Tumor Extension and Increases Death from Metastasis
99	84	Imhof	Tan	Ophthalmology	1996	Quantification of Orbital and Mid Facial Growth Retardation After Megavoltage External Beam Irradiation in Children with Retinoblastoma
100	84	Wilson	Spencer	Clinical genetics	1977	Chromosomal-anomalies in Patients with Retinoblastoma

References

1. Draper, G.J.; Sanders, B.M.; E Kingston, J. Second primary neoplasms in patients with retinoblastoma. *Br. J. Cancer* **1986**, *53*, 661–671, https://doi.org/10.1038/bjc.1986.110.
2. Wong, F.L. Cancer incidence after retinoblastoma. Radiation dose and sarcoma risk. *JAMA* **1997**, *278*, 1262–1267, https://doi.org/10.1001/jama.278.15.1262.
3. Yunis, J.J.; Ramsay, N. Retinoblastoma and Subband Deletion of Chromosome 13. *Arch. Pediatr. Adolesc. Med.* **1978**, *132*, 161–163, https://doi.org/10.1001/archpedi.1978.02120270059012.
4. Kivelä, T. The epidemiological challenge of the most frequent eye cancer: retinoblastoma, an issue of birth and death. *Br. J. Ophthalmol.* **2009**, *93*, 1129–1131, https://doi.org/10.1136/bjo.2008.150292.
5. Abramson, D.H.; Ellsworth, R.M.; Kitchin, F.D.; Tung, G. Second Nonocular Tumors in Retinoblastoma Survivors. *Ophthalmology* **1984**, *91*, 1351–1355, https://doi.org/10.1016/s0161-6420(84)34127-6.
6. Linn Murphree, A. Intraocular Retinoblastoma: the Case for a New Group Classification. *Ophthalmol. Clin. N. Am.* **2005**, *18*, 41–53, https://doi.org/10.1016/j.ohc.2004.11.003.
7. Shields, C.L.; Mashayekhi, A.; Au, A.K.; Czyz, C.; Leahey, A.; Meadows, A.T.; Shields, J.A. The International Classification of Retinoblastoma Predicts Chemoreduction Success. *Ophthalmology* **2006**, *113*, 2276–2280, https://doi.org/10.1016/j.ophtha.2006.06.018.
8. Murphree, A.L.; Villablanca, J.G.; Deegan, W.F.; Sato, J.K.; Malogolowkin, M.; Fisher, A.; Parker, R.; Reed, E.; Gomer, C.J. Chemotherapy Plus Local Treatment in the Management of Intraocular Retinoblastoma. *Arch. Ophthalmol.* **1996**, *114*, 1348–1356, https://doi.org/10.1001/archopht.1996.01100140548005.
9. Marees, T.; van Leeuwen, F.; de Boer, M.; Imhof, S.; Ringens, P.; Moll, A. Cancer mortality in long-term survivors of retinoblastoma. *Eur. J. Cancer* **2009**, *45*, 3245–3253, https://doi.org/10.1016/j.ejca.2009.05.011.
10. Knudson, A.G.; Hethcote, H.W.; Brown, B.W. Mutation and childhood cancer: a probabilistic model for the incidence of retinoblastoma. *Proc. Natl. Acad. Sci.* **1975**, *72*, 5116–5120, https://doi.org/10.1073/pnas.72.12.5116.
11. Knudson, A.G.; Meadows, A.T.; Nichols, W.W.; Hill, R. Chromosomal Deletion and Retinoblastoma. *New Engl. J. Med.* **1976**, *295*, 1120–1123, https://doi.org/10.1056/nejm197611112952007.
12. Shields, C.L.; De Potter, P.; Himelstein, B.P.; Shields, J.A.; Meadows, A.T.; Maris, J.M. Chemoreduction in the Initial Management of Intraocular Retinoblastoma. *Arch. Ophthalmol.* **1996**, *114*, 1330–1338, https://doi.org/10.1001/archopht.1996.01100140530002.
13. Tombran-Tink, J.; Johnson, L.V. Neuronal differentiation of retinoblastoma cells induced by medium conditioned by human RPE cells. *Investig. Opthalmology Vis. Sci.* **1989**, *30*.
14. Kingston, J.E. Results of Combined Chemotherapy and Radiotherapy for Advanced Intraocular Retinoblastoma. *Arch. Ophthalmol.* **1996**, *114*, 1339–43, https://doi.org/10.1001/archopht.1996.01100140539004.
15. Broaddus, E.; Topham, A.; Singh, A.D. Incidence of retinoblastoma in the USA: 1975-2004. *Br. J. Ophthalmol.* **2008**, *93*, 21–23, https://doi.org/10.1136/bjo.2008.138750.
16. Abramson, D.H.; Frank, C.M. Second nonocular tumors in survivors of bilateral retinoblastoma: A possible age effect on radiation-related risk. *Ophthalmology* **1998**, *105*, 573–580, https://doi.org/10.1016/s0161-6420(98)94006-4.
17. Zampetti-Bosseler, F.; Scott, D. Cell Death, Chromosome Damage and Mitotic Delay in Normal Human, Ataxia Telangiectasia and Retinoblastoma Fibroblasts after X-irradiation. *Int. J. Radiat. Biol. Relat. Stud. Physics, Chem. Med.* **1981**, *39*, 547–558, https://doi.org/10.1080/09553008114550651.
18. Jensen, R.D.; Miller, R.W. Retinoblastoma: Epidemiologic Characteristics. *New Engl. J. Med.* **1971** *285*, 307–311, https://doi.org/10.1056/nejm197108052850602.
19. Shields, C.L.; A Shields, J. Retinoblastoma management: advances in enucleation, intravenous chemoreduction, and intra-arterial chemotherapy. *Curr. Opin. Ophthalmol.* **2010**, *21*, 203–212, https://doi.org/10.1097/icu.0b013e328338676a.

20. Knudson A.G . Retinoblastoma: a prototypic hereditary neoplasm. *Semin Oncol*. **1978,** 5,57–60. https://pubmed.ncbi.nlm.nih.gov/635597/
21. Friedman, D.L.; Himelstein, B.; Shields, C.L.; Shields, J.A.; Needle, M.; Miller, D.; Bunin, G.R.; Meadows, A.T. Chemoreduction and Local Ophthalmic Therapy for Intraocular Retinoblastoma. *J. Clin. Oncol.* **2000**, *18*, 12–12, https://doi.org/10.1200/jco.2000.18.1.12.
22. McFall, R.C.; Sery, T.W.; Makadon, M. Characterization of a new continuous cell line derived from a human retinoblastoma.*Cancer Res* **1977**, *37*.
23. Yamane, T.; Kaneko, A.; Mohri, M. The technique of ophthalmic arterial infusion therapy for patients with intraocular retinoblastoma. *Int. J. Clin. Oncol.* **2004**, *9*, 69–73, https://doi.org/10.1007/s10147-004-0392-6.
24. Khelfaoui F, Validire P, Auperin A, et al. Histopathologic risk factors in retinoblastoma: a retrospective study of 172 patients treated in a single institution. *Cancer***. 1996**,*77*:1206–1213. https://pubmed.ncbi.nlm.nih.gov/8635145/
25. Lee, C.T.; Bilton, S.D.; Famiglietti, R.M.; Riley, B.A.; Mahajan, A.; Chang, E.L.; Maor, M.H.; Woo, S.Y.; Cox, J.D.; Smith, A.R. Treatment planning with protons for pediatric retinoblastoma, medulloblastoma, and pelvic sarcoma: How do protons compare with other conformal techniques?. *Int. J. Radiat. Oncol.* **2005**, *63*, 362–372, https://doi.org/10.1016/j.ijrobp.2005.01.060.
26. The American Brachytherapy Society - Ophthalmic Oncology Task Force. The American Brachytherapy Society consensus guidelines for plaque brachytherapy of uveal melanoma and retinoblastoma. *Brachytherapy* **2014**, *13*, 1–14, doi:10.1016/j.brachy.2013.11.008.
27. Shields C.L., Shields J.A., Baez K., et al .Optic nerve invasion of retinoblastoma. Metastatic potential and clinical risk factors. *Cancer*. **1994**,*3*, 692–698. https://pubmed.ncbi.nlm.nih.gov/8299091/
28. Honavar, S.; Singh, A.D.; Shields, C.L.; Meadows, A.T.; Demirci, H.; Cater, J.; Shields, J.A. Postenucleation Adjuvant Therapy in High-Risk Retinoblastoma. *Arch. Ophthalmol.* **2002**, *120*, 923–931, https://doi.org/10.1001/archopht.120.7.923.
29. Shields, C.L.; Shields, J.A.; Baez, K.A.; Cater, J.; De Potter, P.V. Choroidal invasion of retinoblastoma: metastatic potential and clinical risk factors. *Br. J. Ophthalmol.* **1993**, *77*, 544–548, https://doi.org/10.1136/bjo.77.9.544.
30. Shields, C.L.; Honavar, S.G.; Meadows, A.T.; A Shields, J.; Demirci, H.; Singh, A.; Friedman, D.L.; Naduvilath, T.J. Chemoreduction plus focal therapy for retinoblastoma: factors predictive of need for treatment with external beam radiotherapy or enucleation. *Am. J. Ophthalmol.* **2002**, *133*, 657–664, https://doi.org/10.1016/s0002-9394(02)01348-x.
31. Kivelä, T. Trilateral Retinoblastoma: A Meta-Analysis of Hereditary Retinoblastoma Associated With Primary Ectopic Intracranial Retinoblastoma. *J. Clin. Oncol.* **1999**, *17*, 1829–1829, https://doi.org/10.1200/jco.1999.17.6.1829.
32. Abramson, D.H.; Frank, C.M.; Dunkel, I.J. A phase I/II study of subconjunctival carboplatin for intraocular retinoblastoma. *Ophthalmology* **1999**, *106*, 1947–1950, https://doi.org/10.1016/s0161-6420(99)90406-2.
33. Kopelman, J.E.; McLean, I.W.; Rosenberg, S.H. Multivariate Analysis of Risk Factors for Metastasis in Retinoblastoma Treated by Enucleation. *Ophthalmology* **1987**, *94*, 371–377, https://doi.org/10.1016/s0161-6420(87)33436-0.
34. Chintagumpala, M.; Chevez-Barrios, P.; Paysse, E.A.; Plon, S.E.; Hurwitz, R. Retinoblastoma: Review of Current Management. *Oncol.* **2007**, *12*, 1237–1246, https://doi.org/10.1634/theoncologist.12-10-1237.
35. Gombos, D.S.; Hungerford, J.; Abramson, D.H.; Kingston, J.; Chantada, G.; Dunkel, I.J.; Antoneli, C.B.; Greenwald, M.; Haik, B.G.; Leal, C.A.; et al. Secondary Acute Myelogenous Leukemia in Patients with Retinoblastoma: Is Chemotherapy a Factor?. *Ophthalmology* **2007**, *114*, 1378–1383, https://doi.org/10.1016/j.ophtha.2007.03.074.
36. Abramson, D.H.; Frank, C.M.; Susman, M.; Whalen, M.P.; Dunkel, I.J.; Boyd, N.W. Presenting signs of retinoblastoma. *J. Pediatr.* **1998**, *132*, 505–508, https://doi.org/10.1016/s0022-3476(98)70028-9.
37. Shields, C.L.; Shields, J.A. Basic understanding of current classification and management of retinoblastoma. *Curr. Opin. Ophthalmol.* **2006**, *17*, 228–234, https://doi.org/10.1097/01.icu.0000193079.55240.18.
38. Abramson, D.H.; Shields, C.L.; Munier, F.L.; Chantada, G.L. Treatment of Retinoblastoma in 2015. *JAMA Ophthalmol* **2015**, *133*, 1341–1347, https://doi.org/10.1001/jamaophthalmol.2015.3108.
39. Chantada, G.; Doz, F.; Antoneli, C.B.; Grundy, R.; Stannard, F.C.; Dunkel, I.J.; Grabowski, E.; Leal-Leal, C.; Rodríguez-Galindo, C.; Schvartzman, E.; et al. A proposal for an international retinoblastoma staging system. *Pediatr. Blood Cancer* **2005**, *47*, 801–805, https://doi.org/10.1002/pbc.20606.
40. Shields, C.L.; Shields, J.A.; Needle, M.; De Potter, P.; Kheterpal, S.; Hamada, A.; Meadows, A.T. Combined chemoreduction and adjuvant treatment for intraocular retinoblastoma. *Ophthalmology* **1997**, *104*, 2101–2111, https://doi.org/10.1016/s0161-6420(97)30053-0.

41. Sastre, X.; Chantada, G.L.; Doz, F.; Wilson, M.W.; de Davila, M.T.G.; Rodríguez-Galindo, C.; Chintagumpala, M.; Chévez-Barrios, P. Proceedings of the Consensus Meetings From the International Retinoblastoma Staging Working Group on the Pathology Guidelines for the Examination of Enucleated Eyes and Evaluation of Prognostic Risk Factors in Retinoblastoma. *Arch. Pathol. Lab. Med.* **2009**, *133*, 1199–1202, https://doi.org/10.5858/133.8.1199.
42. Sanders, B.M.; Draper, G.J.; Kingston, J.E.Retinoblastoma in Great Britain 1969-80: Incidence, treatment, and survival.*Br. J. Ophthalmol.* **1988**, *72*, 576–583, https://doi.org/10.1136/bjo.72.8.576.
43. Lalande, M.; Dryja, T.P.; Schreck, R.R.; Shipley, J.; Flint, A.; Latt, S.A. Isolation of human chromosome 13-specific DNA sequences cloned from flow sorted chromosomes and potentially linked to the retinoblastoma locus. *Cancer Genet. Cytogenet.* **1984**, *13*, 283–295, https://doi.org/10.1016/0165-4608(84)90073-6.
44. Tamboli, A. The Incidence of Retinoblastoma in the United States: 1974 Through 1985. *Arch. Ophthalmol.* **1990**, *108*, 128–32, https://doi.org/10.1001/archopht.1990.01070030134045.
45. Hethcote, H.W.; Knudson, A.G. Model for the incidence of embryonal cancers: application to retinoblastoma. *Proc. Natl. Acad. Sci.* **1978**, *75*, 2453–2457, https://doi.org/10.1073/pnas.75.5.2453.
46. Broaddus, E.; Topham, A.; Singh, A.D. Survival with retinoblastoma in the USA: 1975-2004. *Br. J. Ophthalmol.* **2008**, *93*, 24–27, https://doi.org/10.1136/bjo.2008.143842.
47. Draper, G.J.; Sanders, B.M.; Brownbill, P.A.; Hawkins, M.M. Patterns of risk of hereditary retinoblastoma and applications to genetic counselling. *Br. J. Cancer* **1992**, *66*, 211–219, https://doi.org/10.1038/bjc.1992.244.
48. Canturk, S.; Qaddoumi, I.; Khetan, V.; Ma, Z.; Furmanchuk, A.; Antoneli, C.B.G.; Sultan, I.; Kebudi, R.; Sharma, T.; Rodriguez-Galindo, C.; et al. Survival of retinoblastoma in less-developed countries impact of socioeconomic and health-related indicators. *Br. J. Ophthalmol.* **2010**, *94*, 1432–1436, https://doi.org/10.1136/bjo.2009.168062.
49. Ts'O, M.O.; Fine, B.S.; Zimmerman, L.E. The Nature of Retinoblastoma. Ii. Photoreceptor Differentiation: An Electron Microscopic Study. *Am. J. Ophthalmol.* **1970**, *69*, 350–359, https://doi.org/10.1016/0002-9394(70)92264-6.
50. Abramson D.H., Ellsworth R.M., Zimmerman L.E. Nonocular cancer in retinoblastoma survivors. *Trans Sect Ophthalmol Am Acad Ophthalmol Otolaryngol.* **1976** *;81*:454–457. https://DOI.org/10.1002/cncr.28387
51. Rodriguez-Galindo, C.; Wilson, M.W.; Haik, B.G.; Merchant, T.E.; Billups, C.A.; Shah, N.; Cain, A.; Langston, J.; Lipson, M.; Kun, L.E.; et al. Treatment of Intraocular Retinoblastoma With Vincristine and Carboplatin. *J. Clin. Oncol.* **2003**, *21*, 2019–2025, https://doi.org/10.1200/jco.2003.09.103.
52. Moll, A.C.; Imhof, S.M.; Meeteren, A.Y.S.-V.; Kuik, D.J.; Hofman, P.; Boers, M. Second primary tumors in hereditary retinoblastoma: a register-based study, 1945–1997: Is there an age effect on radiation-related risk?. *Ophthalmology* **2001**, *108*, 1109–1114, https://doi.org/10.1016/s0161-6420(01)00562-0.
53. Shields, C.L.; Bianciotto, C.G.; Jabbour, P.; Griffin, G.C.; Ramasubramanian, A.; Rosenwasser, R.; Shields, J.A. Intra-arterial Chemotherapy for Retinoblastoma. *JAMA Ophthalmol* **2011**, *129*, 1407–1415, https://doi.org/10.1001/archophthalmol.2011.151.
54. Pendergrass, T.W.; Davis, S. Incidence of Retinoblastoma in the United States. *Arch. Ophthalmol.* **1980**, *98*, 1204–1210, https://doi.org/10.1001/archopht.1980.01020040056003.
55. Ts'O, M.O.; Zimmerman, L.E.; Fine, B.S. The Nature of Retinoblastoma. I. Photoreceptor Differentiation: A Clinical and Histopathologic Study. *Am. J. Ophthalmol.* **1970**, *69*, 339–349, https://doi.org/10.1016/0002-9394(70)92263-4.
56. Chantada, G.; Fandiño, A.; Manzitti, J.; Urrutia, L.; Schvartzman, E. Late diagnosis of retinoblastoma in a developing country. *Arch. Dis. Child.* **1999**, *80*, 171–174, https://doi.org/10.1136/adc.80.2.171.
57. Shields, C.L. Plaque radiotherapy for retinoblastoma Long-term tumor control and treatment complications in 208 tumors. *Ophthalmology* **2001**, *108*, 2116–2121, https://doi.org/10.1016/s0161-6420(01)00797-7.
58. Kaliki, S.; Shields, C.L.; Rojanaporn, D.; Al-Dahmash, S.; McLaughlin, J.P.; Shields, J.A.; Eagle, R.C. High-Risk Retinoblastoma Based on International Classification of Retinoblastoma: Analysis of 519 Enucleated Eyes. *Ophthalmology* **2013**, *120*, 997–1003, https://doi.org/10.1016/j.ophtha.2012.10.044.
59. Abramson, D.H.; Beaverson, K.; Sangani, P.; Vora, R.A.; Lee, T.C.; Hochberg, H.M.; Kirszrot, J.; Ranjithan, M. Screening for Retinoblastoma: Presenting Signs as Prognosticators of Patient and Ocular Survival. *Pediatrics* **2003**, *112*, 1248–1255, https://doi.org/10.1542/peds.112.6.1248.

60. Abramson, D.H.; Ronner, H.J.; Ellsworth, R.M. Second Tumors in Nonirradiated Bilateral Retinoblastoma. *Am. J. Ophthalmol.* **1979**, *87*, 624–627, https://doi.org/10.1016/0002-9394(79)90293-9.
61. Shields, C.L.; Fulco, E.M.; Arias, J.D.; Alarcon, C.; Pellegrini, M.; Rishi, P.; Kaliki, S.; Bianciotto, C.G.; A Shields, J. Retinoblastoma frontiers with intravenous, intra-arterial, periocular, and intravitreal chemotherapy. *Eye* **2012**, *27*, 253–264, https://doi.org/10.1038/eye.2012.175.
62. Shields, C.L. Factors Predictive of Recurrence of Retinal Tumors, Vitreous Seeds, and Subretinal Seeds Following Chemoreduction for Retinoblastoma. *Arch. Ophthalmol.* **2002**, *120*, https://doi.org/10.1001/archopht.120.4.460.
63. Messmer, E.P.; Heinrich, T.; Höpping, W.; de Sutter, E.; Havers, W.; Sauerwein, W. Risk Factors for Metastases in Patents with Retinoblastoma. *Ophthalmology* **1991**, *98*, 136–141, https://doi.org/10.1016/s0161-6420(91)32325-x.
64. Moll, A.C.; Kuik, D.J.; Bouter, L.; Otter, W.D.; Bezemer, P.D.; Koten, J.W.; Imhof, S.M.; Kuyt, B.P.; Tan, K.E.W.P. Incidence and survival of retinoblastoma in the Netherlands: a register based study 1862–1995. *Br. J. Ophthalmol.* **1997**, *81*, 559–562, https://doi.org/10.1136/bjo.81.7.559.
65. Nagasawa, H.; Little, J.B. Comparison of kinetics of X-ray-induced cell killing in normal, ataxia telangiectasia and hereditary retinoblastoma fibroblasts. *Mutat. Res. Mol. Mech. Mutagen.* **1983**, *109*, 297–308, https://doi.org/10.1016/0027-5107(83)90054-4.
66. Devesa, S.S. The Incidence of Retinoblastoma. *Am. J. Ophthalmol.* **1975**, *80*, 263–265, https://doi.org/10.1016/0002-9394(75)90143-9.
67. Jakobiec, F.A.; Tso, M.O.M.; Zimmerman, L.E.; Danis, P. Retinoblastoma and intracranial malignancy. *Cancer* **1977**, *39*, 2048–2058, https://doi.org/10.1002/1097-0142(197705)39:5<2048::aid-cncr2820390522>3.0.co;2-9.
68. Shields, C.L.; A Shields, J. Recent Developments in the Management of Retinoblastoma. *J. Pediatr. Ophthalmol. Strabismus* **1999**, *36*, 8–9, https://doi.org/10.3928/0191-3913-19990101-04.
69. Leander, C.; Fu, L.C.; Peña, A.; Howard, S.C.; Rodriguez-Galindo, C.; Wilimas, J.A.; Ribeiro, R.C.; Haik, B. Impact of an education program on late diagnosis of retinoblastoma in Honduras. *Pediatr. Blood Cancer* **2006**, *49*, 817–819, https://doi.org/10.1002/pbc.21052.
70. Shields, C.L.; Santos, M.C.M.; Diniz, W.; Gündüz, K.; Mercado, G.; Cater, J.R.; Shields, J.A. Thermotherapy for Retinoblastoma. *Arch. Ophthalmol.* **1999**, *117*, 885–893, https://doi.org/10.1001/archopht.117.7.885.
71. Squire, J.; Gallie, B.; Phillips, R.A. A detailed analysis of chromosomal changes in heritable and non-heritable retinoblastoma. *Hum. Genet.* **1985**, *70*, 291–301, https://doi.org/10.1007/bf00295364.
72. Benedict, W.F.; Banerjee, A.; Mark, C.; Murphree, A. Nonrandom chromosomal changes in untreated retinoblastomas. *Cancer Genet. Cytogenet.* **1983**, *10*, 311–333, https://doi.org/10.1016/0165-4608(83)90090-0.
73. de Graaf, P.; on behalf of the European Retinoblastoma Imaging Collaboration (ERIC); Göricke, S.; Rodjan, F.; Galluzzi, P.; Maeder, P.; Castelijns, J.A.; Brisse, H.J. Guidelines for imaging retinoblastoma: imaging principles and MRI standardization. *Pediatr. Radiol.* **2011**, *42*, 2–14, https://doi.org/10.1007/s00247-011-2201-5.
74. Jr, R.C.E. High-Risk Features and Tumor Differentiation in Retinoblastoma: A Retrospective Histopathologic Study. *Arch. Pathol. Lab. Med.* **2009**, *133*, 1203–1209, https://doi.org/10.5858/133.8.1203.
75. Magramm, I.; Abramson, D.H.; Ellsworth, R.M. Optic Nerve Involvement in Retinoblastoma. *Ophthalmology* **1989**, *96*, 217–222, https://doi.org/10.1016/s0161-6420(89)32910-1.
76. Shields, C.L.; Lally, S.E.; Leahey, A.M.; Jabbour, P.M.; Caywood, E.H.; Schwendeman, R.; Shields, J.A. Targeted retinoblastoma management. *Curr. Opin. Ophthalmol.* **2014**, *25*, 374–385, https://doi.org/10.1097/icu.0000000000000091.
77. Wilson M.G., Towner J.W., Fujimoto A. Retinoblastoma and D-chromosome deletions. *Am J Hum Genet.* **1973**,*1*:57–61. https://pubmed.ncbi.nlm.nih.gov/4119334/
78. Lennox, E.L.; Draper, G.J.; Sanders, B.M. Retinoblastoma: a study of natural history and prognosis of 268 cases. *BMJ* **1975**, *3*, 731–734, https://doi.org/10.1136/bmj.3.5986.731.
79. Abramson, D.H. Retinoblastoma in the 20th Century: Past Success and Future Challenges The Weisenfeld Lecture. *Investig. Opthalmology Vis. Sci.* **2005**, *46*, 2684–2691, https://doi.org/10.1167/iovs.04-1462.
80. Chantada, G.L.; Dunkel, I.J.; de Dávila, M.T.G.; Abramson, D.H. Retinoblastoma patients with high risk ocular pathological features: who needs adjuvant therapy?. *Br. J. Ophthalmol.* **2004**, *88*, 1069–1073, https://doi.org/10.1136/bjo.2003.037044.
81. Abramson, D.H.; Marr, B.P.; Dunkel, I.; Brodie, S.; Zabor, E.C.; Driscoll, S.J.; Gobin, Y.P. Intra-arterial chemotherapy for retinoblastoma in eyes with vitreous and/or subretinal seeding: 2-year results. *Br. J. Ophthalmol.* **2011**, *96*, 499–502, https://doi.org/10.1136/bjophthalmol-2011-300498.
82. Weichselbaum, R.R.; Nove, J.; Little, J.B. X-ray sensitivity of diploid fibroblasts from patients with hereditary or sporadic retinoblastoma. *Proc. Natl. Acad. Sci.* **1978**, *75*, 3962–3964, https://doi.org/10.1073/pnas.75.8.3962.

83. Shields, J.A.; Augsburger, J.J. Current approaches to the diagnosis and management of retinoblastoma. *Surv. Ophthalmol.* **1981**, *25*, 347–372, https://doi.org/10.1016/0039-6257(81)90072-2.
84. Beck, M.N.; Balmer, A.; Dessing, C.; Pica, A.; Munier, F. First-Line Chemotherapy With Local Treatment Can Prevent External-Beam Irradiation and Enucleation in Low-Stage Intraocular Retinoblastoma. *J. Clin. Oncol.* **2000**, *18*, 2881–2887, https://doi.org/10.1200/jco.2000.18.15.2881.
85. A Bedford, M.; Bedotto, C.; A Macfaul, P. Retinoblastoma. A study of 139 cases. *Br. J. Ophthalmol.* **1971**, *55*, 19–27, https://doi.org/10.1136/bjo.55.1.19.
86. .Sethi R.V., Shih H.A., Yeap B.Y., et al. Second nonocular tumors among survivors of retinoblastoma treated

with contemporary photon and proton radiotherapy. *Cancer*. **2014**,*1*,126–133. DOI: 10.1002/cncr.28387.

87. Qaddoumi, I.; Bass, J.; Wu, J.; Billups, C.A.; Wozniak, A.W.; Merchant, T.E.; Haik, B.G.; Wilson, M.W.; Rodriguez-Galindo, C. Carboplatin-Associated Ototoxicity in Children With Retinoblastoma. *J. Clin. Oncol.* **2012**, *30*, 1034–1041, https://doi.org/10.1200/jco.2011.36.9744.
88. Namouni, F.; Doz, F.; Tanguy, M.; Quintana, E.; Michon, J.; Pacquement, H.; Bouffet, E.; Gentet, J.; Plantaz, D.; Lutz, P.; et al. High-dose chemotherapy with carboplatin, etoposide and cyclophosphamide followed by a haematopoietic stem cell rescue in patients with high-risk retinoblastoma: a SFOP and SFGM study. *Eur. J. Cancer* **1997**, *33*, 2368–2375, https://doi.org/10.1016/s0959-8049(97)10019-3.
89. Stannard, C.; Lipper, S.; Sealy, R.; Sevel, D. Retinoblastoma: correlation of invasion of the optic nerve and choroid with prognosis and metastases. *Br. J. Ophthalmol.* **1979**, *63*, 560–570, https://doi.org/10.1136/bjo.63.8.560.
90. Sparkes, R.S.; Muller, H.; Klisak, I.; Abram, J.A. Retinoblastoma with 13q- Chromosomal Deletion Associated with Maternal Paracentric Inversion of 13q. *Science* **1979**, *203*, 1027–1029, https://doi.org/10.1126/science.424728.
91. Uusitalo, M.S.; Van Quill, K.R.; Scott, I.U.; Matthay, K.K.; Murray, T.G.; O'Brien, J.M. Evaluation of chemoprophylaxis in patients with unilateral retinoblastoma with high-risk features on histopathologic examination. *Arch. Ophthalmol.* **2001**, *119*.
92. Schvartzman, E.; Chantada, G.; Fandiño, A.; De Dávila, M.T.; Raslawski, E.; Manzitti, J. Results of a stage-based protocol for the treatment of retinoblastoma. *J. Clin. Oncol.* **1996**, *14*, 1532–1536, https://doi.org/10.1200/jco.1996.14.5.1532.
93. Moll A.C., Imhof S.M., Bouter .LM,et al. Second primary tumors in patients with hereditary retinoblastoma: a register-based follow-up study, 1945–1994. *Int J Cancer*. **1996** *4*, 515–519. DOI: 10.1002/(SICI)1097-0215(19960807)67:4<515::AID-IJC9>3.0.CO;2-V.
94. Doz, F.; Khelfaoui, F.; Mosseri, V.; Validire, P.; Quintana, E.; Michon, J.; M.D., L.D.; Schlienger, P.; M.D., S.N.; Vielh, P.; et al. The role of chemotherapy in orbital involvement of retinoblastoma. The experience of a single institution with 33 patients. *Cancer* **1994**, *74*, 722–732, https://doi.org/10.1002/1097-0142(19940715)74:2<722::aid-cncr2820740228>3.0.co;2-h.
95. Yu, C.-L.; Tucker, M.A.; Abramson, D.H.; Furukawa, K.; Seddon, J.M.; Stovall, M.; Fraumeni, J.F.; Kleinerman, R. Cause-Specific Mortality in Long-Term Survivors of Retinoblastoma. *Gynecol. Oncol.* **2009**, *101*, 581–591, https://doi.org/10.1093/jnci/djp046.
96. Derkinderen, D.J.; Koten, J.W.; Nagelkerke, N.J.D.; Tan, K.E.W.P.; Beemer, F.A.; Otter, W.D. Non-ocular cancer in patients with hereditary retinoblastoma and their relatives. *Int. J. Cancer* **1988**, *41*, 499–504, https://doi.org/10.1002/ijc.2910410405.
97. Egbert, P.R.; Donaldson, S.S.; Moazed, K.; Rosenthal, A.R. Visual Results and Ocular Complications Following Radiotherapy for Retinoblastoma. *Arch. Ophthalmol.* **1978**, *96*, 1826–1830, https://doi.org/10.1001/archopht.1978.03910060338008.
98. Zhao, J.; Dimaras, H.; Massey, C.; Xu, X.; Huang, D.; Li, B.; Chan, H.S.; Gallie, B.L. Pre-Enucleation Chemotherapy for Eyes Severely Affected by Retinoblastoma Masks Risk of Tumor Extension and Increases Death From Metastasis. *J. Clin. Oncol.* **2011**, *29*, 845–851, https://doi.org/10.1200/jco.2010.32.5332.
100. Imhof, S.M.; Mourits, M.P.; Hofman, P.; Zonneveld, F.W.; Schipper, J.; Moll, A.C.; Tan, K.E. Quantification of Orbital and Mid-facial Growth Retardation after Megavoltage External Beam Irradiation in Children with Retinoblastoma. *Ophthalmology* **1996**, *103*, 263–268, https://doi.org/10.1016/s0161-6420(96)30706-9.
101. Wilson, M.G.; Ebbin, A.J.; Towner, J.W.; Spencer, W.H. Chromosomal anomalies in patients with retinoblastoma. *Clin. Genet.* **2008**, *12*, 1–8, https://doi.org/10.1111/j.1399-0004.1977.tb00894.x.
